# Predicting invasive breast cancer versus DCIS in different age groups

**DOI:** 10.1186/1471-2407-14-584

**Published:** 2014-08-11

**Authors:** Mehmet US Ayvaci, Oguzhan Alagoz, Jagpreet Chhatwal, Alejandro Munoz del Rio, Edward A Sickles, Houssam Nassif, Karla Kerlikowske, Elizabeth S Burnside

**Affiliations:** Information Systems and Operations Management, University of Texas at Dallas, 800 W Campbell Rd, SM 33, Richardson, TX 75080-3021 USA; Industrial & Systems Engineering, University of Wisconsin, 1513 University Avenue, Madison, WI 53706 USA; Department of Health Services Research, MD Anderson Cancer Center at University of Texas, 1400 Pressler Street, Unit 1444, Houston, TX 77098 USA; Department of Radiology, University of Wisconsin School of Medicine and Public Health, E3/311 Clinical Science Center, 600 Highland Ave., Madison, WI 53792-3252 USA; Department of Radiology, University of California, San Francisco, CA 94143 USA; Department of Computer Science, University of Wisconsin, Madison, WI 53706 USA; Departments of Medicine and Epidemiology and Biostatistics, University of California, San Francisco, CA 94143 USA

**Keywords:** Mammography, Logistic models, Breast neoplasms, Overdiagnosis, Biopsy, Aging

## Abstract

**Background:**

Increasing focus on potentially unnecessary diagnosis and treatment of certain breast cancers prompted our investigation of whether clinical and mammographic features predictive of invasive breast cancer versus ductal carcinoma in situ (DCIS) differ by age.

**Methods:**

We analyzed 1,475 malignant breast biopsies, 1,063 invasive and 412 DCIS, from 35,871 prospectively collected consecutive diagnostic mammograms interpreted at University of California, San Francisco between 1/6/1997 and 6/29/2007. We constructed three logistic regression models to predict the probability of invasive cancer versus DCIS for the following groups: women ≥ 65 (older group), women 50–64 (middle age group), and women < 50 (younger group). We identified significant predictors and measured the performance in all models using area under the receiver operating characteristic curve (AUC).

**Results:**

The models for older and the middle age groups performed significantly better than the model for younger group (AUC = 0.848 vs, 0.778; p = 0.049 and AUC = 0.851 vs, 0.778; p = 0.022, respectively). Palpability and principal mammographic finding were significant predictors in distinguishing invasive from DCIS in all age groups. Family history of breast cancer, mass shape and mass margins were significant positive predictors of invasive cancer in the older group whereas calcification distribution was a negative predictor of invasive cancer (i.e. predicted DCIS). In the middle age group—mass margins, and in the younger group—mass size were positive predictors of invasive cancer.

**Conclusions:**

Clinical and mammographic finding features predict invasive breast cancer versus DCIS better in older women than younger women. Specific predictive variables differ based on age.

**Electronic supplementary material:**

The online version of this article (doi:10.1186/1471-2407-14-584) contains supplementary material, which is available to authorized users.

## Background

The literature reflects that breast cancer has a unique pathophysiology based on age. Younger patients have a higher frequency of estrogen receptor-negative, higher-grade tumors and older patients have a higher rate of estrogen receptor-positive, low-grade tumors [1–5]. Evidence in the literature also demonstrates that mammography features using standardized descriptors (found in the Breast Imaging Reporting and Data System—BI-RADS) can predict the histology of breast cancer [6, 7]. Several studies have demonstrated the feasibility of predicting the probability of invasive breast cancer versus DCIS using patient characteristics and mammographic findings [8, 9], by treating age groups uniformly. Our goal was to show that the inherent age-based differences in breast cancer pathophysiology will affect the predictive ability of these models, resulting in differential accuracy and distinct predictive features based on age.

We were motivated to investigate this question because of the increasing interest in addressing the potentially unnecessary diagnosis and treatment of certain breast cancers. Ductal carcinoma in situ (DCIS), a non-obligate precursor to subsequent invasive breast cancer [10, 11], may remain indolent for sufficiently long that a woman dies of other causes, a phenomenon referred to as overdiagnosis [12, 13]. An extremely valuable cohort of 28 DCIS cases inadvertently treated by biopsy alone revealed that 39% of these women developed invasive breast cancer in the same quadrant, same breast over a median follow-up of 31 years, 5 of whom (45) died from metastatic disease [10]. The lengthy natural history of some cases of DCIS implies that women with a limited life expectancy are less likely to benefit from treatment on a population level. However, to date, the medical community does not know which women are likely to benefit from diagnosis and treatment, thus DCIS will continue to be treated as the standard of care outside of clinical trials.

This clinical challenge has substantial public health impact. The age-adjusted incidence rate of ductal carcinoma in situ (DCIS) between 1973 and 2000 increased from 4.3 to 32.7 per 100,000 women-years, an increase of 660% [14], the majority of cases detected on mammographic screening [15]. While incidence increased in all age groups, the increased rate of DCIS was most notable in women > 50 [16]. The 2009 National Institutes of Health (NIH) consensus conference on DCIS highlighted the need for data to improve our understanding of and management decisions around this increasingly common diagnosis [17]. Two particularly important components of this “call to action” include: 1) gaining a better understanding of the characteristics of DCIS versus invasive cancer in distinct patient populations, for example, women of different ages, that may someday guide optimal management based on expected natural history of disease and 2) discovering unique features of DCIS in these same populations in order to inform prospective identification and enable personalization of care.

Thus, the specific purpose of this study was to confirm the hypothesis that age-related differences exist when discriminating invasive breast cancer from DCIS. In addition, we aimed to discover the clinical and mammographic features that are differentially predictive based on age.

## Methods

### Patients

The University of California, San Francisco (UCSF) Institutional Review Board approved this Health Insurance Portability and Accountability Act-compliant study. In addition, they waived the requirement for informed consent because there were no patient identifiers associated with the data, thereby minimizing any risk (particularly confidentiality risk). Our initial dataset consisted of 146,198 consecutive mammograms with 35,871 diagnostic exams that were prospectively collected between 1/6/1997 to 6/9/2007 from UCSF and were interpreted by 13 radiologists. This facility used eight analog mammography units during the collection of the data. Mammography reports were generated during routine clinical practice, using a semi-structured format recording patient characteristics, breast density, and the principal mammographic finding for abnormal examinations. Additional details describing the findings were dictated in free text by the interpreting radiologist. Mammography features were based on the BI-RADS lexicon, which consists of descriptors and final assessment categories that standardize mammography reporting [18].

**Figure 1:**
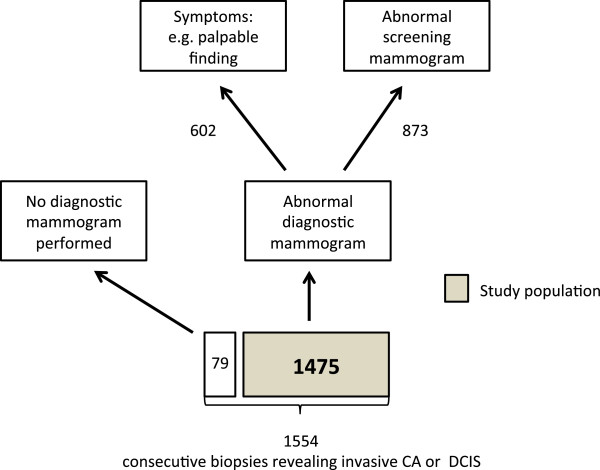
**Patient population derived from consecutive image guided biopsies revealing cancer.**

We used pathology results from biopsy (within this same timeframe) as our reference standard to determine if breast cancer cases were invasive or DCIS. We labeled biopsies that revealed both invasive cancer and DCIS as invasive. We found a total of 4,081 biopsies of which, 1,554 revealed invasive cancer or DCIS. We matched each biopsy with a preceding diagnostic mammography exam less than 90 days prior to biopsy. We excluded 79 biopsies that did not have corresponding diagnostic mammograms, leaving 1,475 biopsies eligible for study, performed on 1,384 women (Figure 1).

We populated mammographic variables according to the BI-RADS lexicon in two ways. Patient characteristics and mammographic descriptors reported in structured format were exported directly. Mammographic descriptors contained in the free text reports were extracted via a natural language processing (NLP) algorithm previously developed and evaluated [19]. A total of 10 variables were available in structured format and six variables were extracted via the NLP code (Table 1). In the structured part of our database, we labeled all missing variables as “missing.” In the rest of this manuscript, the term “biopsy” refers to the entire record including clinical/demographic factors, mammographic findings (from the associated diagnostic mammogram), and the pathologic finding from the biopsy: invasive cancer or DCIS.

**Table 1:** List of structured and extracted variables*

Structured	Variables extracted using NLP
• Age	• Calcification distribution
• Family history (of breast cancer)†	• Calcification morphology¥
• Personal history (of breast cancer)	• Mass margins
• Prior surgery‡	• Mass shape
• Palpable lump	• Architectural distortion
• Breast density	• Focal asymmetric density
• BI-RADS assessment	
• Indication for exam if diagnostic	
• Principal mammography findingΨ	
• Mass size	

*These variables were used as input to the stepwise regression to produce the models for older and younger women.

†Defined as family history of breast cancer (Minor = one or more relatives more distant than first-degree relatives, Strong = one first-degree relative with unilateral postmenopausal breast cancer, Very Strong = more than one first-degree relative with unilateral postmenopausal breast cancer, one first-degree relative with bilateral breast cancer, or one first-degree with premenopausal breast cancer).

‡Defined as prior breast surgery of any kind.

ΨPrincipal mammographic finding: architectural distortion, calcifications, asymmetry (one view), focal asymmetry (two views), developing asymmetry, mass, single dilated duct, both calcifications and something else.

¥To overcome low frequency categories, features are grouped into high probability malignancy, intermediate and typically benign categories, as described in the Breast Imaging and Reporting Data System (BI-RADS) lexicon [18].

### Statistical analysis

We designated women ≥ 65 as the older group, women 50–64 as the middle group, and women < 50 as the younger group. We developed three separate multiple-predictor logistic regression models one for each age group, using R [20]. For interested readers, we constructed a fourth model for the whole biopsy population (including all ages) using the same methodology (Additional file 1). Each model included clinical and mammographic predictor variables (from Table 1) and a binary outcome variable (invasive/DCIS). We defined positive as invasive cancer and negative as DCIS. We used backward/forward stepwise regression with Akaike information criterion (AIC) to obtain our models [21]. The Wald chi-square statistic was used to assess the significance of model predictors. All p-values were from two-sided tests with a significance level of 0.05. Due to limited number of pair-wise comparison, p-values were not adjusted for multiple testing (see Additional file 2 for further details of the statistical analysis).

To evaluate the performance of our models, we used a modified leave-one-out cross validation, a process that provided an estimated probability of invasive cancer for each biopsy. Biopsies assigned a probability above a given threshold were, by definition, predicted to be invasive cancer. Biopsies assigned a probability below that threshold were, by definition, predicted to be DCIS. Using this procedure, we calculated the number of true positives (invasive prediction and invasive outcome), false positives (invasive prediction and DCIS outcome), true negatives (DCIS prediction and DCIS outcome), and false negatives (DCIS prediction and invasive outcome) at all possible thresholds between 0 and 100%. We then used probability estimates and outcomes to create receiver operating characteristics (ROC) curves and calculate the area under the curves (AUC). We compared AUC values using methods appropriate for unpaired and uncorrelated ROC curves using a nonparametric approach [22].

## Results

### Data

Of the 1,475 biopsies analyzed, 1,063 revealed invasive breast cancer diagnoses and 412 revealed DCIS. Of the 1384 included patients, 86 had multiple biopsies; 81 patients were biopsied twice and 5 patients were biopsied three times. The age of the subjects ranged from 27 to 97 with mean 43.1 for the younger group, 56.6 for middle age group, and 74.5 for the older group. We found that the proportion of DCIS was slightly higher in the younger and middle age groups than the overall proportion with a lower proportion in the older group (Table 2).

**Table 2:** **Proportion of DCIS in each age group**

	Biopsies revealing DCIS	Biopsies revealing invasive carcinoma	Total biopsies	Total patients	DCIS percentage (%) and the 95% confidence interval
**Age < 50**	110	264	374	353	29.4 (25.0,34.2)
**50 < =Age < =64**	170	398	568	538	29.9 (26.3, 33.8)
**Age > =65**	132	401	533	493	24.8 (21.3,28.6)
**Total**	412	1063	1475	1384	27.9 (25.7,30.3)

### Logistic regression models in different age groups

In our models, if a variable is positively correlated with invasive cancer it is also negatively correlated with DCIS (because the outcome variable and the outcomes of all cases are binary: invasive cancer or DCIS). Thus, we will typically summarize our results in terms of the correlation with our positive outcome—invasive cancer. However the converse (the opposite direction correlation with DCIS) will also be mentioned when clinically relevant.

In the model for the older group, presence of a palpable lump (p = 0.013), family history of breast cancer (p = 0.043), principal mammography finding (p < 0.001), mass margins (p < 0.001), and mass shape (p = 0.033) were statistically significant in positively predicting invasive cancer. Calcification distribution (p = 0.008) was also statistically significant but was negatively correlated with invasive cancer (positively correlated with DCIS). Prior surgery (p = 0.132) and focal asymmetric density (p = 0.077) were included by stepwise regression due to their predictive ability of invasive cancer, despite being non-significant. The remaining variables as listed in Table 1 did not improve the AIC of the fitted model, therefore were not included in the final model (Table 3).

**Table 3:** Multivariable model for older group using stepwise regression with AIC criterion*

Risk factor		Beta	Odds ratio	95% CI (Lower -Upper)			p value		
	(Intercept)	−1.16	0.31	0.18	-	0.55		0.000	***
**Palpable lump**							*0.013*		**
	No corresponding palpable mass	0.00	1(referent)						
	Missing	−0.30	0.74	0.05	-	10.55		0.824	
	Corresponding palpable mass	0.80	2.22	1.12	-	4.41		0.022	**
**Family history**							*0.043*		**
	None	0.00	1(referent)						
	Missing	−0.89	0.41	0.13	-	1.32		0.135	
	Strong	−0.32	0.73	0.33	-	1.59		0.422	
	Very strong	1.66	5.24	0.84	-	32.78		0.076	*
**Prior surgery**							*0.132*		
	Not present	0.00	1(referent)						
	Missing	−0.36	0.70	0.07	-	6.82		0.759	
	Present	0.57	1.78	0.99	-	3.17		0.053	*
**Principal mammography finding**							*<0.001*		***
	Calcifications or Single dilated duct	0.00	1(referent)						
	Architectural distortion	20.56	Inf	0.00	-	Inf		0.993	
	Associated calcifications	2.16	8.67	3.39	-	22.14		0.000	***
	Missing	2.10	8.14	3.88	-	17.09		0.000	***
	Asymmetry or Focal asymmetry	2.94	18.87	3.79	-	93.87		0.000	***
	Mass	3.04	20.93	9.20	-	47.65		0.000	***
	Developing asymmetry	2.80	16.45	1.78	-	151.95		0.014	**
**Calcification distribution**							*0.008*		**
	Not present	0.00	1(referent)						
	Linear or Segmental	−3.11	0.04	0.00	-	0.49		0.011	**
	Clustered	−0.69	0.50	0.22	-	1.18		0.113	
	Regional or Scattered	−1.94	0.14	0.01	-	2.83		0.202	
**Mass margins**							*<0.001*		***
	None	0.00	1(referent)						
	Circumscribed	−2.51	0.08	0.01	-	0.45		0.004	***
	Ill-Defined	0.19	1.21	0.46	-	3.20		0.703	
	Obscured	0.10	1.10	0.11	-	11.31		0.935	
	Spiculated	28.70	Inf	0.00	-	Inf		0.983	
**Mass shape**							*0.033*		**
	None	0.00	1(referent)						
	Irregular	1.91	6.78	0.78	-	58.79		0.083	*
	Lobular or Oval	−0.13	0.87	0.24	-	3.16		0.838	
	Round	−15.53	0.00	0.00	-	Inf		0.987	
**Focal asymmetric density**							*0.077*		*
	Not present	0.00	1(referent)						
	Present	1.63	5.10	0.54	-	47.77		0.154	

The model is presented in the order of inclusion into the model.

*Asterisks denote the level of significance such that: ***p-value < 0.001; **p-value < 0.05, and *p-value <0.1.

“Inf” (short for infinity) is inserted at places where the data for the corresponding variable is sparsely populated and produces a very high and unstable odds ratio.

In the model for middle age group, presence of a palpable lump (p < 0.001), principal mammography finding (p < 0.001), and mass margins (p < 0.001) were significant in predicting and positively correlated with invasive cancer. In addition, prior surgery (p = 0.050) and mass shape (p = 0.080) were included due to their predictive ability of invasive cancer, despite being non-significant (Table 4).

**Table 4:** Multivariable model for the middle group using stepwise regression with AIC criterion*

Risk factor		Beta	Odds ratio	95% CI (Lower -Upper)			p value		
	(Intercept)	-1.367	0.255	0.16	-	0.41		<0.001	***
**Principal mammography finding**							*<0.001*		***
	Calcifications or Single dilated duct	0	1(referent)						
	Architectural distortion	18.123	Inf	0	-	Inf		0.979	
	Associated calcifications	1.911	6.757	3.04	-	15.03		<0.001	***
	Missing	0.562	1.754	0.95	-	3.23		0.072	*
	Asymmetry or Focal asymmetry	2.212	9.13	1.83	-	45.5		0.007	***
	Mass	2.81	16.604	7.54	-	36.55		<0.001	***
	Developing asymmetry	18.049	Inf	0	-	Inf		0.991	
**Palpable lump**							*<0.001*		***
	No corresponding palpable mass	0	1(referent)						
	Missing	1.01	2.74	0.68	-	11.1		0.158	
	Corresponding palpable mass	1.2	3.322	1.91	-	5.79		<0.001	***
**Mass margins**							*<0.001*		***
	None	0	1(referent)						
	Circumscribed	0.529	1.697	0.14	-	20.5		0.677	
	Ill-Defined	0.24	1.272	0.31	-	5.24		0.74	
	Obscured	16.662	Inf	0	-	Inf		0.991	
	Spiculated	2.6	13.463	3.03	-	59.76		0.001	***
**Prior surgery**							*0.05*		**
	Not present	0	1(referent)						
	Missing	0.729	2.074	0.97	-	4.42		0.059	*
	Present	0.627	1.871	1.07	-	3.28		0.029	**
**Mass shape**							*0.08*		*
	None	0	1(referent)						
	Irregular	2.114	8.28	0.96	-	71.31		0.054	*
	Lobular or Oval	0.728	2.072	0.36	-	11.88		0.414	
	Round	16.123	Inf	0	-	Inf		0.997	

The model is presented in the order of inclusion into the model.

*Asterisks denote the level of significance such that: *** p-value < 0.001; **p-value < 0.05, and * p-value <0.1.

“Inf” (short for infinity) is inserted at places where the data for the corresponding variable is sparsely populated and produces a very high and unstable odds ratio.

In the model for younger women, presence of a palpable lump (p < 0.001), principal mammography finding (p < 0.001), and mass size (p = 0.047) were significant in predicting and positively correlated with invasive cancer. In addition, architectural distortion (p = 0.063) and mass shape (p = 0.090) were included due to their predictive ability of invasive cancer, despite being non-significant (Table 5).

**Table 5:** Multivariable model for younger group using stepwise regression with AIC criterion*

Risk factor		Beta	Odds ratio	95% CI (Lower -Upper)			p value		
	(Intercept)	−0.64	0.53	0.35	-	0.8		0.002	***
**Palpable lump**							*0*		***
	No corresponding palpable mass	0	1(referent)						
	Missing	−0.68	0.51	0.16	-	1.6		0.246	
	Corresponding palpable mass	1.21	3.36	1.79	-	6.32		0	***
**Principal mammography finding**							*0*		***
	Calcifications or Single dilated duct	0	1(referent)						
	Architectural distortion	1.95	7.05	0.75	-	65.98		0.087	*
	Associated calcifications	1.58	4.85	1.87	-	12.55		0.001	***
	Missing	1.02	2.76	1.34	-	5.7		0.006	***
	Asymmetry or Focal asymmetry	1.86	6.41	1.26	-	32.64		0.025	**
	Mass	2.74	15.51	4.97	-	48.35		0	***
	Developing asymmetry	16.5	Inf	0	-	Inf		0.997	
**Architectural distortion**							*0.063*		*
	Not present	0	1(referent)						
	Present	1.78	5.91	0.67	-	52.13		0.11	
**Mass shape**							*0.09*		*
	None	0	1(referent)						
	Irregular	15.83	Inf	0	-	Inf		0.986	
	Lobular or Oval	0.09	1.1	0.23	-	5.21		0.787	
	Round	−19.53	0	0	-	Inf		0.996	
**Mass size**							*0.047*		*
	None	0	1(referent)						
	20-Oct	−0.97	0.38	0.03	-	4.61		0.447	
	20-50	1.7	5.47	1.17	-	25.69		0.031	**
	<10	−0.58	0.56	0.19	-	1.63		0.287	
	> = 50	−0.58	0.56	0.14	-	2.25		0.413	

The model is presented in the order of inclusion into the model.

*Asterisks denote the level of significance such that: *** p-value < 0.001; **p-value < 0.05, and * p-value <0.1.

“Inf” (short for infinity) is inserted at places where the data for the corresponding variable is sparsely populated and produces a very high and unstable odds ratio.

For completeness, we also built a forth logistic regression model for the whole biopsy population (Additional file 1). In this model, the presence of a palpable lump (p < 0.001), principal mammographic finding (p < 0.001), mass margins (p < 0.001), and mass shape (p = 0.001) were significant in predicting and positively correlated with invasive cancer. Three non-significant variables positively correlated with invasive cancer: family history of breast cancer (p = 0.080), BI-RADS assessment (p = 0.13), architectural distortion (p = 0.15): and one non-significant variable negatively correlated with invasive cancer: calcification distribution (p = 0.080) were included by stepwise regression due to their predictive ability (Additional file 1: Table S1).

**Figure 2:**
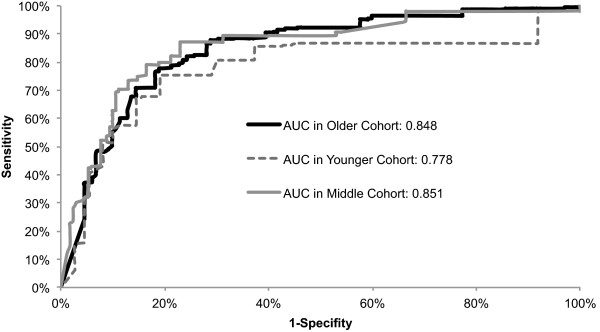
**ROC curves for age specific models.** Graph shows receiver operating characteristic (ROC) curves constructed from predictions from multivariable logistic regression models for older, middle, and younger group. AUC refers to area under the ROC curve and SE refers to standard error.

We compared the performance of our models in discriminating between invasive cancer and DCIS using AUC values (Figure 2). The models for older and the middle age groups performed significantly better than the model for younger group (AUC = 0.848 vs, 0.778; p = 0.049 and AUC = 0.851 vs, 0.778; p = 0.022, respectively). The AUC difference between the model for older group and the middle group was not statistically significant (p = 0.803).

**Figure 3:**
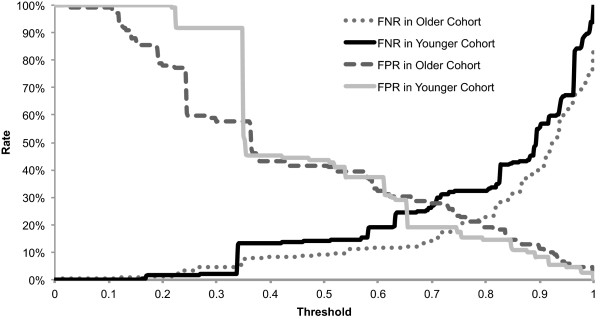
**Misclassification rates of models for older versus younger group at all possible thresholds.** False negative rate (FNR) and false positive rate (FPR) for two of the age-based models: the older group (dashed lines) and the younger group (solid lines), are graphed for all threshold levels.

Next, we plotted the misclassification rates for two models (for the younger and older groups) at all possible thresholds between 0-100%, above which the biopsy was predicted to be invasive (Figure 3). Clinically, misclassifying invasive cancer as DCIS is a more serious error (defined as a false negative) than misclassifying DCIS as an invasive cancer (defined as a false positive). The false negative rate was lower for the older group at almost all threshold levels of risk when compared to the younger group. In other words, the model for older group performed better than that for the younger group in terms of accurately predicting invasive cancer. The false positive rate was also better for the older group at lower threshold levels but appeared equivalent to or slightly worse than the younger group at higher threshold levels.

## Discussion

Our logistic regression models demonstrate that differentiation of invasive cancer from DCIS using clinical and mammographic features is more accurate in the older (≥65) and middle age (50–64) groups than in the younger group (<50). We found that presence of a palpable lump and the principal mammographic finding type were statistically significant predictors of invasive cancer versus DCIS in all three models. However, we did find variable combinations that uniquely predict invasive cancer based on age. Family history, mass shape, and mass margins were significant positive predictors of invasive cancer in the older age group whereas calcification distribution was negatively associated with invasive cancer (positively associated with DCIS). Mass margin was a significant predictor of invasive cancer in the middle age group. Mass size was a significant predictor in the younger group. These age-based combinations are different from the significant variables identified using a single model for the whole group (Additional file 1), which included presence of a palpable lump, principal mammographic finding, mass margins, and mass shape.

Thus, we validate our original hypothesis that the ability to differentiate invasive cancer from DCIS based on clinical and mammography features depends on age. We posit several possible explanations for this age dependence. First, since we know that the pathophysiology of invasive breast cancer differs with age [1–4], perhaps this disease difference manifests in distinct mammographic appearance that allows better prediction in older versus younger women [23, 24]. Second, superior predictive performance in the older group may be related to the higher sensitivity and positive predictive value of mammography (usually attributed to decreasing breast density) in this population [25, 26]. In other words, radiologists may be able to identify and characterize findings predictive of invasive versus DCIS with more accuracy and precision in older women. Importantly, age, menopausal status, breast density, distinct breast cancer pathophysiology, and the accuracy of mammography, are interrelated and may contribute in complex ways to superior predictive ability in the older group. Third, increasing breast cancer incidence seen with advancing age [27] may also partially explain the differential performance that we identify. A larger number of cancers in our middle and older group may provide more statistical power to enable demonstration of better performance as compared to the younger group.

Our work reinforces prior research showing that both clinical and mammography features can contribute to predicting the risk of invasive disease versus DCIS considering all age groups together [8, 9, 28, 29]. However, we demonstrate that distinct variables are uniquely predictive of invasive cancer in different age groups. Clinical variables like prior surgery may have high predictive ability in only older group because this variable has more time to accumulate in older group possibly lending more power to this predictor. Of note, in our results, a very strong family history of breast cancer is more positively correlated with invasive cancer than DCIS in the older but not the younger age group. This appears counter to the finding in recent literature that breast cancer risk associated with family history actually decreases with age when comparing women with and without breast cancer [30]. Our result is particularly intriguing. Despite strong evidence that the risk of all types of breast cancer related to family history decreases with age, the risk of invasive cancer compared to DCIS may actually increase with age. This finding deserves further study.

Masses found on mammography were significant predictors in all age groups. However, certain mass descriptors predicted invasive cancer in only one group. Mass shape was a significant predictor of invasive cancer in the older group, mass margin was a significant predictor in the older and middle groups, and mass size was a significant predictor in the younger group. These results suggest that margins and shape may be more difficult to reliably assess in younger women due to high breast density. Breast density has previously been shown to be a strong risk factor for both invasive cancer and DCIS compared to women without cancer [31]. Our results are consistent with this finding in that we did not find breast density to be a stronger predictor of invasive versus DCIS in our study nor was it differentially predictive based on age.

Because the rationale for our study was to test whether clinical and mammography variables were differentially predictive of invasive breast cancer versus DCIS based on age, we do not claim that our predictive model would be appropriate for use in clinical practice. Nevertheless, our study is an important step in demonstrating that predicting invasive versus in situ breast disease appears to be possible and superior for older and middle age women as compared to younger women. Prospective prediction of invasive versus in situ breast cancer will require more sophisticated and accurate models, inclusion of consecutive cases of both benign and malignant diagnoses, and development of improved predictors, possibly molecular markers that confer invasive risk [32].

Our predictive models are limited by the unavoidable challenge of clinical data that is inherently imperfect. We believe we were justified in assuming a high performance of NLP extraction of free text predictors based on the fact that these algorithms [19, 33] have been shown to perform well previously in a similar task. However, our dataset does not include some of the breast cancer risk factors that are well established albeit with moderate impact on risk such as body mass index [8, 29]. Inclusion of such variables in larger databases may improve prediction accuracy.

Several study design decisions, though necessary to validate our specific hypothesis, may limit the generalizability of our results to other scientific questions. For example, our decision to exclude benign cases and include only the malignant cases in this study precludes us from using our models for prospective risk prediction. However, we did not intend to create a predictive model to be used prior to biopsy but rather to demonstrate age based differences in the differentiation of invasive cancer from DCIS as well as identify predictors that differ based on age. Our decision to group women into three age groups was a compromise weighing several considerations. First, these age groups are convenient because they reflect the usual age grouping in incidence and mortality reporting [34]. Second, these cut-offs split the data roughly into tertiles. Third, we hoped this grouping strategy might balance sample size constraints with a clear demarcation between pre-menopausal (the younger) and post-menopausal (the older) age groups. The literature demonstrates that the median age at natural menopause is 52.54 years in a multi-ethnic population in the US [35, 36]. Our results for the middle age group are consistent with this threshold because these women (ranging in age from 50 to 64) are likely predominantly comprised of post-menopausal women. That is why the middle age group was more similar to the older (undoubtedly post-menopausal) group in terms of risk factors for invasive breast cancer versus DCIS than they were to the younger group. We recognize that earlier work is wary of assignment of women into specific age groups with abrupt cut point (most commonly done at age 50) because outcomes do not suddenly change at these specified thresholds [37]. Of note, age, included as a continuous variable in our logistic regression (see Additional file 1), was not a significant predictor and thus does not shed further light on this relationship. Analysis of the interactions between smaller intervals of age in this discrimination task would be interesting; however, larger data sets would be required in order to provide the power to observe these differences.

## Conclusion

We are encouraged that our logistic regression model documented age-based differences in the discrimination between invasive cancer and DCIS, performing best in older age groups. Unique age-based predictive variables provide a first clue as to what clinical and mammographic features may be valuable as we start to contemplate risk-based screening and diagnosis of breast cancers most likely to cause harm. Additional research will be crucial for further elucidation of the reasons for the age-based differences in predictive variables and their interactions with age, menopausal status, breast cancer pathophysiology, and mammography accuracy. Elucidating these relationships will likely be a step toward ultimately improving physicians’ ability to prospectively distinguish invasive breast cancer and DCIS in the pursuit of personalized and optimal care.

## Electronic supplementary material

Additional file 1: **Model for all women and trial of age as a predictor variable** [38]**.** (DOC 78 KB)

Additional file 2: **Advanced statistical methods** [39]**.** (DOC 26 KB)
