# Supplementary material for: Predicting invasive breast cancer versus DCIS in different age groups
Source: BMC Cancer. 2014 Aug 11;14:584. doi: 10.1186/1471-2407-14-584 (PMC4138370; doi:10.1186/1471-2407-14-584)
Supplement: Supplementary file 1 — Additional file 1: Model for all women and trial of age as a predictor variable [38]. (DOC 78 KB) [file 12885_2013_4770_MOESM1_ESM.doc]

*Additional file 1 - Model for All Women and Trial of Age as a Predictor Variable*

***Methods***

*Methods for model creation*

Initially, we created a single logistic regression model for all women including age as a continuous or categorical predictor variable. While the logistic regression model for all women would normally need to include interaction terms for exploring age-dependent effects, we chose not to include interaction variables to prevent the risk of overfitting the model to a small data set. More specifically, there are a total of additional 15 or 15 × 2 = 30 interaction terms (depending on whether age is considered continuous or categorical, respectively) which would stretch the minimum of 10 DCIS biopsies per predictor advocated by a well-known rule of thumb for logistic regression . Our approach potentially allows for more stable parameter estimates, but precludes formal testing of effects across age groups.

***Results***

*Trial of Age as a Predictor Variable*

Since we fitted a main effects (additive) model with age as a predictor variable, we would expect that age would be included as a significant variable in our logistic regression model if, in fact, age influences the ability of imaging features to discriminate between invasive cancer and DCIS. However, age was not a significant predictor and therefore did not show up in the stepwise regression result when modeled as a continuous variable or categorical variable using the same predefined age groups.

In the model for all women, four variables were statistically significant in predicting invasive cancer versus DCIS and four variables were included by stepwise regression due to their predictive ability, despite being non-significant (Table S1). The remaining variables did not improve the AIC of the fitted model, therefore were not included in the final model. Specifically, age was not a significant variable when we compared the stepwise regression model with and without age. The model for all women had an AUC performance of 0.842.

**Table S1. Multivariable model for all women using stepwise regression with AIC Criterion*. The model is presented in the order of inclusion into the model.**

| **Risk Factor** | | **Beta** | **Odds Ratio** | **95% CI (Lower -Upper)** | | | **p value** | |  |
| --- | --- | --- | --- | --- | --- | --- | --- | --- | --- |
|  | (Intercept) | -0.84 | 0.43 | 0.14 | - | 1.32 |  | 0.141 |  |
| **Palpable Lump** | |  |  |  |  |  | **<0.001** | | *** |
|  | No Corresponding Palpable Mass | 0 | 1(referent) |  |  |  |  |  |  |
|  | Missing | -0.03 | 0.97 | 0.48 | - | 1.93 |  | 0.926 |  |
|  | Corresponding Palpable Mass | 1.03 | 2.81 | 1.99 | - | 3.96 |  | <0.001 | *** |
| **Family History** | |  |  |  |  |  | **0.08** |  | * |
|  | None | 0 | 1(referent) |  |  |  |  |  |  |
|  | Missing | -0.2 | 0.82 | 0.43 | - | 1.58 |  | 0.553 |  |
|  | Strong | -0.27 | 0.76 | 0.49 | - | 1.18 |  | 0.218 |  |
|  | Very Strong | 0.65 | 1.92 | 1.03 | - | 3.57 |  | 0.039 | ** |
| **BI-RADS Assessment** | |  |  |  |  |  | **0.13** |  |  |
|  | 0 | 0 | 1(referent) |  |  |  |  |  |  |
|  | 4 | -0.04 | 0.96 | 0.32 | - | 2.88 |  | 0.945 |  |
|  | 5 | 0.34 | 1.41 | 0.46 | - | 4.37 |  | 0.551 |  |
| **Principal Mammographic Finding** | |  |  |  |  |  | **<0.001** | | *** |
|  | Calcifications or Single Dilated Duct | 0 | 1(referent) |  |  |  |  |  |  |
|  | Architectural Distortion | 4.35 | 77.76 | 10.11 | - | 597.86 |  | <0.001 | *** |
|  | Associated Calcifications | 1.59 | 4.9 | 2.98 | - | 8.05 |  | <0.001 | *** |
|  | Missing | 1.14 | 3.14 | 2.14 | - | 4.59 |  | <0.001 | *** |
|  | Asymmetry or Focal Asymmetry | 2.48 | 11.9 | 4.84 | - | 29.25 |  | <0.001 | *** |
|  | Mass | 2.57 | 13.08 | 8.19 | - | 20.89 |  | <0.001 | *** |
|  | Developing Asymmetry | 3.06 | 21.42 | 2.72 | - | 168.42 |  | 0.004 | *** |
| **Architectural Distortion** | |  |  |  |  |  | **0.15** |  |  |
|  | Not Present | 0 | 1(referent) |  |  |  |  |  |  |
|  | Present | 0.5 | 1.64 | 0.82 | - | 3.28 |  | 0.16 |  |
| **Mass Margins** | |  |  |  |  |  | **<0.001** | | *** |
|  | None | 0 | 1(referent) |  |  |  |  |  |  |
|  | Circumscribed | -0.9 | 0.41 | 0.13 | - | 1.29 |  | 0.126 |  |
|  | Ill-Defined | 0.27 | 1.31 | 0.66 | - | 2.63 |  | 0.442 |  |
|  | Obscured | 1.23 | 3.41 | 0.4 | - | 29.28 |  | 0.264 |  |
|  | Spiculated | 2.48 | 11.97 | 3.69 | - | 38.87 |  | <0.001 | *** |
| **Mass Shape** | |  |  |  |  |  | **0.001** |  | *** |
|  | None | 0 | 1(referent) |  |  |  |  |  |  |
|  | Irregular | 2.09 | 8.06 | 1.89 | - | 34.34 |  | 0.005 | *** |
|  | Lobular or Oval | 0.1 | 1.1 | 0.48 | - | 2.53 |  | 0.817 |  |
|  | Round | -1.45 | 0.23 | 0.02 | - | 2.55 |  | 0.234 |  |
| **Calcification Distribution** | |  |  |  |  |  | **0.08** |  | * |
|  | Not Present | 0 | 1(referent) |  |  |  |  |  |  |
|  | Linear or Segmental | -1.1 | 0.33 | 0.13 | - | 0.87 |  | 0.025 | ** |
|  | Clustered | -0.22 | 0.8 | 0.52 | - | 1.23 |  | 0.315 |  |
|  | Regional or Scattered | -0.67 | 0.51 | 0.08 | - | 3.18 |  | 0.474 |  |

*Asterisks denote the level of significance such that: *** p-value<0.001; **p-value<0.05, and * p-value <0.1.

“Inf” (short for infinity) is inserted at places where the data for the corresponding variable is sparsely populated and produces a very high and unstable odds ratio.
