# Supplementary material for: Predicting invasive breast cancer versus DCIS in different age groups
Source: BMC Cancer. 2014 Aug 11;14:584. doi: 10.1186/1471-2407-14-584 (PMC4138370; doi:10.1186/1471-2407-14-584)
Supplement: Supplementary file 2 — Additional file 2: Advanced statistical methods [39]. (DOC 26 KB) [file 12885_2013_4770_MOESM2_ESM.doc]

Additional file 2 – Advanced Statistical Methods

*Correlation Effects*

In order to determine the most appropriate statistical method, we did some preliminary data analysis. In particular, we investigated the potential correlation issues. Only 6% of the patients had multiple biopsies in our database and this may potentially be a source of correlation. Though the percentage of patients with multiple biopsies was small, we explored the correlation effects due to including multiple biopsies per subject by conducting both random effects logistic regression and logistic regression analysis using generalized estimating equations, assuming equal correlation for all biopsies . Including within-patient correlation revealed no substantial effect on our models. We also explored standard multiple-predictor logistic regression with and without interaction effects. We then examined the competing models for coefficient magnitude and significance. If these differences were found to be small, then the model of choice would be the standard logistic regression model. The use of interaction effects in the logistic regression model would depend on the significance of interaction effects and the sparsity of information in two way tables.

When we explored the correlation effects by comparing the competing models described above, we found only slight differences between the coefficients and significance levels of all predictors. Also, the vast majority of pair-wise correlations among the predictors were small (less than 0.65) and the results from the stepwise fits were stable, as determined by arriving at the same final model despite changes in the starting model or the lower and upper scope of candidate models. This analysis revealed that the standard logistic regression was sufficient. We also explored interaction effects in our stepwise analysis. We found that the sparse information in the two way tables between predictors made interaction terms difficult to estimate and interpret. Hence, we decided to use standard logistic regression without interaction terms.

*Building Logistic Regression Model*

We built our models using the AIC criterion for stepwise regression. AIC measures the relative goodness of fit where the model with a smaller AIC value is preferred. The inclusion threshold for change in AIC is equivalent to P<0.157 for confounders with one degree of freedom. We set the null model as both the starting and lower bound model, and the main effects model including all predictors in Table 1 as the upper bound model. The model resulting from stepwise regression can include both significant and non-significant predictors since variables are chosen based on their effect on the AIC which does not depend solely on the corresponding p-value.

*Cross validation*

This technique trains the model on all biopsies except the one(s) associated with a given patient and then tests the resultant trained model on the observation(s) associated with the held out patient (i.e. observations not used in training). By using this methodology, we prevented over-optimistic performance estimates stemming from training and testing on the data that belonged to the same patient as it may be a possible source for dependencies.
